# Influence of the Oil Phase and Topical Formulation on the Wound Healing Ability of a Birch Bark Dry Extract

**DOI:** 10.1371/journal.pone.0155582

**Published:** 2016-05-24

**Authors:** Isabel Steinbrenner, Pia Houdek, Simone Pollok, Johanna M. Brandner, Rolf Daniels

**Affiliations:** 1 Department of Pharmaceutical Technology, Eberhard Karls University Tuebingen, Tuebingen, Germany; 2 University Hospital Hamburg Eppendorf, Department of Dermatology & Venerology, Hamburg, Germany; CNRS-University of Toulouse, FRANCE

## Abstract

Triterpenes from the outer bark of birch are known for various pharmacological effects including enhanced wound healing (WH). A birch bark dry extract (TE) obtained by accelerated solvent extraction showed the ability to form oleogels when it is suspended in oils. Consistency of the oleogels and the dissolved amount of triterpenes varies largely with the used oil. Here we wanted to know to what extent different oils and formulations (oleogel versus o/w emulsion) influence WH. Looking at the plain oils, medium-chain triglycerides (MCT) enhanced WH (ca. 1.4-fold), while e.g. castor oil (ca.0.3-fold) or light liquid paraffin (LLP; ca. 0.5-fold) significantly decreased WH. Concerning the respective oleogels, TE-MCT showed no improvement although the solubility of the TE was high. In contrast, the oleogel of sunflower oil which alone showed a slight tendency to impair WH, enhanced WH significantly (ca. 1.6-fold). These results can be explained by release experiments where the release rate of betulin, the main component of TE, from MCT oleogels was significantly lower than from sunflower oil oleogels. LLP impaired WH as plain oil and even though it released betulin comparable to sunflower oil it still results in an overall negative effect of the oleogel on WH. As a further formulation option also surfactant free o/w emulsions were prepared using MCT, sunflower oil and LLP as a nonpolar oil phase. Depending on the preparation method (suspension or oleogel method) the distribution of the TE varied markedly and affected also release kinetics. However, the released betulin was clearly below the values measured with the respective oleogels. Consequently, none of the emulsions showed a significantly positive effect on WH. In conclusion, our data show that the oil used as a vehicle influences wound healing not only by affecting the release of the extract, but also by having its own vehicle effect on wound healing. This is also of importance for other applications where drugs have to be applied in non-polar vehicles because these solvents likely influence the outcome of the experiment substantially.

## Introduction

Biological activity of active ingredients is strongly dependent on their vehicle. (1) Release of active ingredients may be different from different vehicles, (2) the vehicles themselves may influence the microenvironment, e.g. by acting as penetration enhancers and (3) they may directly influence the target cells/ physiological processes. Oils are often used as vehicles, e.g. in wound dressings, irritation studies or in basic science for the application of non-polar organic ligands or inhibitors.

Triterpenes are non-polar biologically active secondary plant metabolites. Their various pharmacological properties like anti-inflammatory, anti-viral, and wound healing effects are well investigated and specified in the literature [1–6]. Also in cancer treatment triterpenes are known as potent agents [7–11]. These substances are widely distributed in plants but only the outer bark of the white barked birches contains up to 34% (w/w) betulin, a pentacyclic, lupan type triterpene with two polar hydroxyl groups located on opposite sides of the molecule [12]. Apart from natural sources, lupan type triterpenes can be obtained by chemical synthesis, especially, betulinic acid (BA) from betulin via an oxidation reaction [13,14]. A well characterized triterpene dry extract from the outer bark of birch (“triterpene extract”, TE) is obtained by accelerated solvent extraction with n-heptane and contains about 80% (w/w) betulin [15]. Further identified main constituents of this dry extract are lupeol (LU), erythrodiol (ER), BA and oleanolic acid (OA) [16] (see also Table 1). In order to make use of the therapeutic potential of the TE, it has been found by chance that this TE forms spreadable thixotropic gels, so called oleogels, when suspended in oils. For an oleogel prepared from TE and sunflower oil it was shown that it has no clear effects on actinic keratosis [17,18], but that it is clearly beneficial for wound healing [2,19,20].

**Table 1:** Chemical composition and physical characteristics of the TE used.

**Chemical composition**	Betulin 81.6%, Lupeol 2.08%, Betulinic acid 3.84%, Erythrodiol 1.05%, Oleanolic acid 0.97%, Metulinic acid methylester 0.52%, unidentified substances 9.94%
**Specific surface area**	42 ± 0.4 m²/g
**Particle size D50%**	5.8 μm

Because of this proven effect of TE oleogel in wound healing we used this extract to test the hypothesis that the oil used as a vehicle substantially influences the effect of active ingredients. Poor solubility of these triterpenes in polar and non-polar solvents are known [16,21] and might limit their therapeutic application due to a poor availability. Only 0.28% (w/w) of the TE are soluble in jojoba oil and < 0.0001% (w/w) are soluble in water [15]. In addition, the oil itself may significantly influence wound healing. We chose wound healing as a model therapeutic system, because the search for effective and less expensive treatment for wounds is still a major aim in research and because a positive effect of TE in sunflower oil on wound healing is already known [2,19,20]. However, the data may also be important for other investigations where drugs have to be applied in oils.

After testing oils and oleogels of TE with the various oils we formed o/w emulsions of the best candidates (sunflower oil and medium-chain triglycerides), because o/w emulsions might serve in a next step as a base for the formulation of o/w foam creams. These foam creams are expected to have superior properties compared to pure oleogels because they can be applied to wounds almost touchless. In addition, these formulations further helped us to elucidate the relationship between oil, active ingredient release and wound healing.

In cases where cytotoxicity is at least an essential part of the active principle, several formulation options to improve the bioactivity of triterpene extracts have been successfully tested, e.g. nano-formulations [22,23] and inclusion complexes with cyclo dextrins [24]. However, in the context of this study we focused on formulation concepts based mainly on the oleogels as they have proved to be promising candidates and cytotoxic effects are counterproductive in wound healing.

The investigated oleogels consisted only of the respective oil phase (diverse oils) and TE, o/w emulsions consisted solely of the respective oil phase (sunflower oil (SFO), medium-chain triglycerides (MCT), light liquid paraffin (LLP)), water, hypromellose (hydroxypropylmethylcellulose, HPMC) and TE. HPMC was chosen as polymeric emulsifier as it allows the formulation of physically stable surfactant free o/w emulsions [25–29] where the emulsifier is expected to not adversely affect living cells [30]. All the formulations are physically stable. In addition to the above mentioned aim to characterize these oleogels and emulsions with respect to wound healing ability, we further investigated the location of TE within the formulation and TE release and discuss the influence of these parameters on the outcome of the wound healing experiments.

## Materials and Methods

### Materials

Triterpene extract from the outer bark of birch, TE, was obtained from Birken GmbH, Niefern-Öschelbronn, Germany (Table 1). Sunflower oil (Henry Lamotte Oils GmbH, Bremen, Germany) medium-chain triglycerides, MCT (Miglyol 812; Sasol Germany GmbH; Hamburg, Germany), and light liquid paraffin (Hansen & Rosenthal, Hamburg, Germany) as well as octyldodecanol (BASF, Ludwigshafen, Germany), peanut oil (Caelo, Hilden, Germany), castor oil (Caelo, Hilden, Germany), almond oil (Caelo, Hilden, Germany), jojoba oil (Gustav Hees, Stuttgart, Germany), isopropylmyristate (BASF, Ludwigshafen, Germany) were used as oil phases. Water was obtained by reverse osmosis (Purelab Option-Q 7/15; Elga Berkenfeld GmbH, Celle, Germany). HPMC, was obtained from Harke Service GmbH (Mühlheim an der Ruhr, Germany). Ethylcellulose was obtained from Dow Chemicals (Schwalbach, Germany)

Release studies were conducted with modified Franz diffusion cells (volume of 12 mL; orifice of 2 cm^2^ Gauer Glas, Püttlingen, Germany) and track-etch polycarbonate membranes 0.4 μm (Whatman, Dassel, Germany).

### Preparation of TE-oleogels

After coarse grinding, TE powder was homogeneously dispersed in the oil phase using an Ultra-Turrax^®^ T 25 (IKA, D-Staufen) at 13,500 rpm for 3 min. TE-oleogels for release experiments were prepared with a TE content of 2.0% and 9.1%, respectively to allow comparison with the o/w formulations. For wound healing experiments, 10% oleogels were used to allow comparison with clinical data [20]. Oleogels were allowed to equilibrate for at least two days at room temperature before further use.

Oleogels did not show any sign of bleeding and changes in consistency during the test period.

### Preparation of o/w emulsions

O/w emulsions consisted of 2.0% TE, 76.0% water, 2.0% HPMC, and 20.0% oil. The o/w emulsions were prepared by two different methods:

#### Method 1 “suspension method”

For this purpose, an aqueous 5% (m/m) TE-suspension was prepared using a colloid mill (Fryma-Koruma, Neuenburg, Germany). HPMC-powder was added to this suspension and the mixture was homogenized using an Ultra-Turrax^®^ T 25 (IKA, D-Staufen) at 13,500 rpm for 3 min and at 24,000 rpm for 1 min. After addition of the oil phase, the emulsion was again homogenized at 13,500 rpm for 2 min and at 24,000 for 1 min.

D50%-values of the droplet size distribution were 4.2 μm (SFO), 4.3 μm (MCT), and 9.1 μm (LLP), respectively.

#### Method 2 “oleogel method”

TE-oleogel was added to an HPMC-gel and homogenized with an Ultra-Turrax^®^ at 13,500 rpm for 3 min and subsequently at 24,000 rpm for 1 min.

D50%-values of the droplet size distribution were 4.5 μm (SFO), 5.4 μm (MCT), and 7.8 μm (LLP), respectively.

Emulsions were stored for at least 3 days at room temperature before further use, to allow the TE to reach partition equilibrium.

Emulsions remained unchanged in their droplet size distribution during the test period.

### Interfacial tension

The interfacial tension was determined using a tensiometer applying the pendant drop method (PAT-1, Sinterface Technologies, Berlin, Germany). The instrument is equipped with a CCD camera and a recording system. Interfacial tension was calculated from the drop shape using the fundamental Laplace equation.

### Dynamic viscosity

Oleogels with medium-chain triglycerides, sunflower oil and light liquid paraffin were characterized rheologically using Physica MCR 501 (Anton Paar Germany GmbH; Ostfildern, Germany). The rotational measurement was conducted using pre-shear and recovery time.

### Raman microscopic characterization of emulsions

Confocal Raman microscopic images were obtained using an alpha 500R confocal Raman microscope (WiTec GmbH, D-Ulm) equipped with a 532 nm excitation laser, UHTS 300 spectrometer and DV401-BV CCD camera. The specific areas were mapped using a 100x objective (numerical aperture 1.25). Details can be seen in the corresponding figures. Colour-coded images were calculated using WiTecProject Suite 4.04 software (WiTec GmbH, D-Ulm).

### Release of betulin

In vitro release studies were conducted using modified Franz diffusion cells (Gauer Glas, D-Püttlingen) with a receptor-volume of 12 mL. Receptor medium was a pH 7.4 phosphate buffer solution (disodium phosphate dodecahydrate; potassium dihydrogen phosphate; Caesar & Loretz, Hilden; Germany) with a supplement of 2-HP-ß-cyclodextrin 10% (m/V) (Kleptose HPB oral grade Roquette Freres, Lestrem, France). Degassed, prewarmed (32°C) receptor medium was filled into Franz diffusion cells. Thereafter, Franz diffusion cells were equipped with polycarbonate membranes 0.4 μm (Whatman, Dassel, Germany). Subsequently, 1.0 g of formulation were spread onto the membranes. Cells were capped with Parafilm. In vitro release experiments were performed at 32°C; stirring speed was 500 rpm. TE was allowed to diffuse over a period of 34 h. Aliquots of 5 mL were withdrawn at 5 time points (6, 10, 24, 30 and 34 h) and the sample volume was replaced by fresh, prewarmed receptor medium. Samples were analyzed by GC. Release rates were calculated by linear regression; release coefficients were calculated by dividing release rates by initial concentration of dissolved betulin in the vehicle according to Eq 1:

$$K_{r}=\frac{J_{ss}}{c_{0}}$$

where K_r_ = release coefficient [mL/cm^2^h], J_ss_ = steady state flux through polycarbonate membranes [μg/cm^2^h] and c_0_ = initial betulin concentration in the formulation [μg/mL] Experiments were performed three times.

### Betulin assay

After adding internal standard solution and processing in the shaker, the sample was washed with ethyl acetat three times. After evaporating and drying, tetrahydrofuran was added and the sample was again evaporated. The backlog was silylated with 60 μL of silylating mixture (Fluka; Buchs, Switzerland) at 80°C for 30 min. Betulin was assayed using a GC method as described by Laszczyk [16].

### Wound healing assay

To assess the wound healing capacity of the oils, oleogels and the TE emulsions, a porcine ex-vivo wound healing model was used as previously described [2,31]. Briefly, pig ears (Schlachterei Hoose, Schleswig-Holstein, Germany) were delivered directly after slaughtering for human consumption to the laboratory, cleaned and disinfected. Then 6 mm punch biopsies were taken from the plicae of the ears and fat and subcutis were removed. Wounds were generated by the removal of the epidermis and upper dermis in a central area of 7.1 mm^2^ and the so formed ex-vivo wound healing model was placed dermis-down on gauze in culture dishes and incubated air-liquid interface with Dulbecco´s modified Eagle´s medium supplemented with hydrocortisone, 2% fetal calf serum, penicillin and streptomycin. 5 μL of the oils/oleogels/emulsions were applied immediately after wounding by using an insulin syringe and the models were incubated for 48 h at 37°C and 5% CO_2_. After shock freezing, cryostat sections of the central parts of the wound healing models—which were identified by using a ruler in the microscope and by checking the total length of the wound during evaluation—were stained with hematoxylin and eosin. Wound healing progress (reepithelialization) was evaluated by measuring the distance between the wound margin and the tip of the regenerated epidermis with an Axiophot II Zeiss microscope and the measurement tool of the Openlab 5.0.2 software (Improvision Coventry, UK). Means of left and right wound margins were calculated. Hair follicles directly at the wound margins, contamination and deep incisions led to the exclusion of wound models or parts of them.

### Statistics

For the comparison of wound healing progress mixed linear models were used and statistical significance was defined as p<0.05.

For the data of the release tests one-sided one factorial analysis of variance (ANOVA) followed by Student-Newman-Keuls test were conducted using p < 0.05.

## Results and Discussion

### Wound healing capacity of TE in different oils

**Fig 1:**
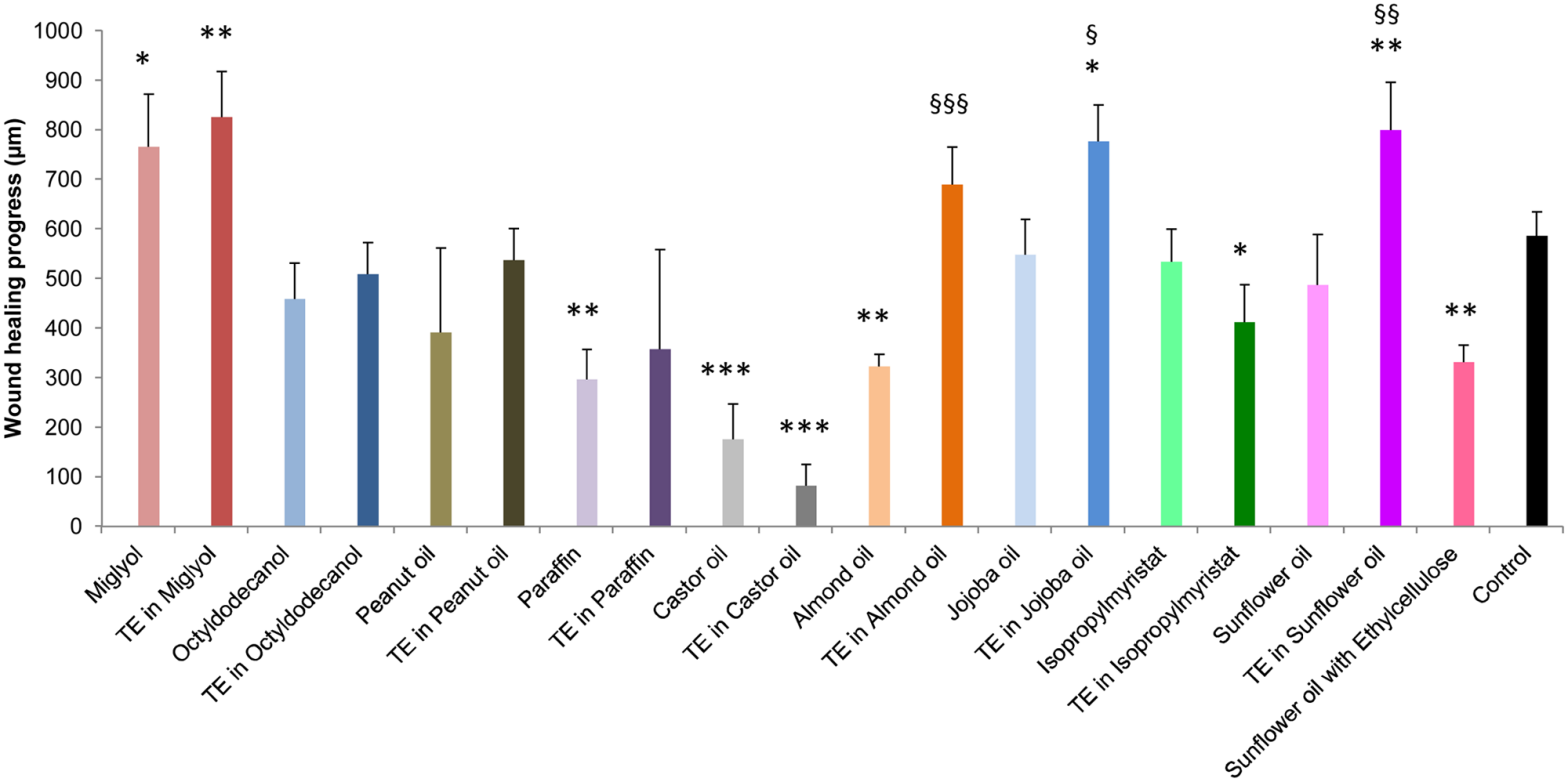
Effect of the various oils and oleogels on wound healing progress in ex-vivo wound healing models after 48 h of treatment. Mean + SEM. * compared to control, § comparison between oil and oleogel. */§. p<0.05, **/§§ p<0.01, ***/§§§ p<0.001. peanut oil, TE in peanut oil, paraffin, TE in paraffin, castor oil, TE in castor oil. n = 4–5; Miglyol (MCT), TE in Miglyol (MCT), octyldodecanol, TE in ocytldodecanol, almond oil, TE in almond oil, jojoba oil, TE in jojoba oil, isopropylmyristate, TE in isopropylmyristate, sunflower oil, TE in sunflower oil, sunflower oil with ethylcellulose: n = 9–14, control: n = 14.

With the aim to investigate the influence of different oils on the outcome of wound healing experiments and to choose the best oil for the generation of a TE containing emulsion, we tested 9 different oils and the respective oleogels in ex vivo wound healing models. While Miglyol (MCT) showed significant wound healing improvement compared to untreated control (1.39±0.15-fold improvement), LLP (0.47±0.09-fold), castor oil (0.28±0.11) and almond oil (0.58±0.09) showed significant impairment of wound healing compared to untreated control. There was also a tendency of wound healing impairment for octyldodecanol, peanut oil, jojoba oil, isopropylmyristate and SFO compared to untreated control (Fig 1).

MCT showed significantly improved wound healing compared to all other oils (Fig 1).

These data clearly show that there is a striking difference concerning the wound healing effects of different oils, some of them resulting in impaired wound healing, others in improved wound healing. This is of special importance, as e.g. castor oil which shows a negative effect, is used in formulations containing balsam of Peru and trypsin (BCT) as emollient and constituent of the vehicle base to treat various kinds of wounds [32–34]. LLP, which also shows a negative effect, is often used in formulations or dressings for the treatment of wounds [35–37]. These data suggest that usage of a different oil in these preparations may be beneficial for wound healing. On the other hand, MCT shows a strikingly good effect on wound healing. To our knowledge, no data concerning its effect on wound healing have been published yet. Thus this is the first report showing the positive effect of this oil on wound healing.

When adding TE to the oil, which means preparing the oleogels, we observed a significant improvement of wound healing for almond oil oleogel compared to almond oil (2.12-fold), jojoba oil oleogel compared to jojoba oil (1.42-fold) and SFO oleogel compared to SFO (1.64-fold). Also compared to SFO with ethylcellulose which we used to mimic the increased viscosity of the oleogel there was a significant increase of wound healing with TE in sunflower oil (2.41-fold) (Fig 1). MCT oleogel (1.56±0.24-fold), SFO oleogel (1.45±0.17-fold) and jojoba oil oleogel (1.39±0.14-fold) also showed significant increase of wound healing compared to untreated controls. However, MCT oleogel did not improve wound healing compared to MCT alone.

TE is an extract from birch bark which has been shown before to be beneficial in wound healing in vitro and ex vivo [2]. It influences inflammation, cell migration, differentiation and barrier formation [2,38]. Interestingly, while there was a significant improvement by addition of TE to SFO, jojoba oil and almond oil, there was no clear improvement by addition of TE e.g. to MCT and LLP. For castor oil and isopropylmyristate, there was even a tendency for impaired wound healing by addition of TE (Fig 1).

This clearly shows that the oil itself and its interaction with TE influence wound healing. The knowledge about this remarkable effect of different oils on the outcome of the experiment may also be important for planning other medical or basic science experiments where oils have to be used to dissolve active ingredients like non-polar inhibitors, agonists or irritants.

### Wound healing capacity of TE in o/w emulsions containing Miglyol and sunflower oil

**Fig 2:**
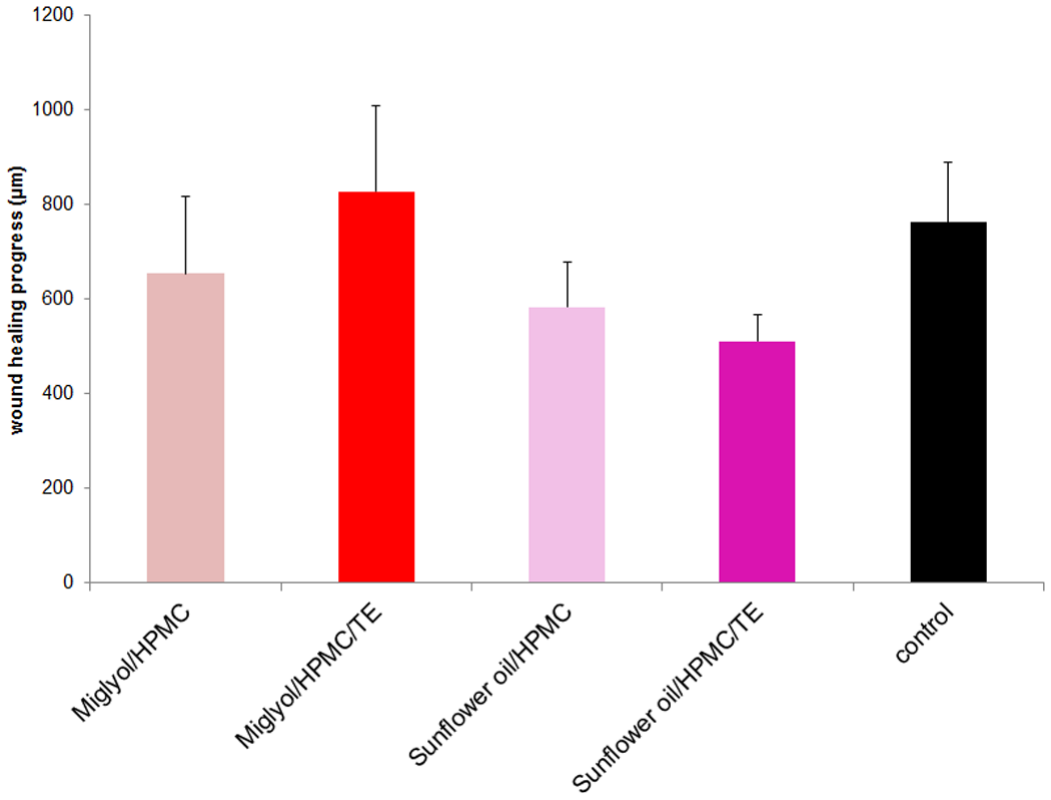
Effect of various emulsions on wound healing progress in ex-vivo wound healing models after 48h of treatment. Emulsions were prepared with method 1. Mean + SEM, n = 4–5.

Because of the positive effect of TE in SFO, which showed improved wound healing compared to its “placebo” (SFO), compared to its “viscosity placebo” (SFO with ethylcellulose) and compared to untreated controls, we decided to further investigate this combination as an oil in water emulsion. Such o/w emulsions are intended to serve in a next step as a base for the formulation of o/w foam creams which are expected to have superior properties compared to pure oleogels because they can be applied to wounds almost touchless and almost painless. We also investigated in comparison TE in MCT, because this oil showed per se a very good wound healing capacity. The emulsions were prepared according to “method 1” (see below). However, we observed that the emulsions exhibited less wound healing capacity than the oils / oleogels (Fig 2). None of the emulsions showed a significantly positive effect compared to the untreated control (Fig 2).

To further elucidate the putative cause for the lower capacity of the oil in water emulsions to improve wound healing compared to the oleogels, we further characterized the TE emulsions with SFO and MCT and their capacity for betulin release. Moreover we investigated formulations with LLP as the oil phase, although we found this oil to affect wound healing negatively (Fig 1). We did so because, in order to elucidate the properties of the formulations, it is expected to add value as it is less polar than the two other oils and can thus change the galenical properties of the formulations significantly.

### Characterization of TE emulsions

We prepared TE emulsions by two different methods (“suspension method” (method 1) and “oleogel method” (method 2) see Materials and Methods). The TE emulsions with the three different oil phases (SFO, MCT, LLP) markedly differ in their polarity, TE solubility, and their respective TE partition coefficients (Table 2). SFO and MCT are oils of medium polarity, whereas LLP is non-polar. The solubility of TE in the three oil phases varies significantly and is at least 500-fold higher than the aqueous solubility of TE [39]. As the concentration of TE in all emulsions exceeds its solubility, a significant amount of TE is suspended in the emulsions. Depending on the preparation method and the affinity of the TE to the respective oil phases its spatial distribution might vary. To elucidate this, confocal Raman microscopic images were taken.

**Table 2:** Interfacial tension, solubility and log P values of TE with different oils.

	refined sunflower oil	medium-chain triglycerides	light liquid paraffin	water
**Interfacial tension**	25.4 mN/m	27.1 mN/m	42.7 mN/m	-
**TE solubility**	4.4 mg/mL	7.9 mg/mL	0.41 mg/mL	0.25 μg/mL
**log P**	4.24	4.50	3.21	-

**Fig 3:**
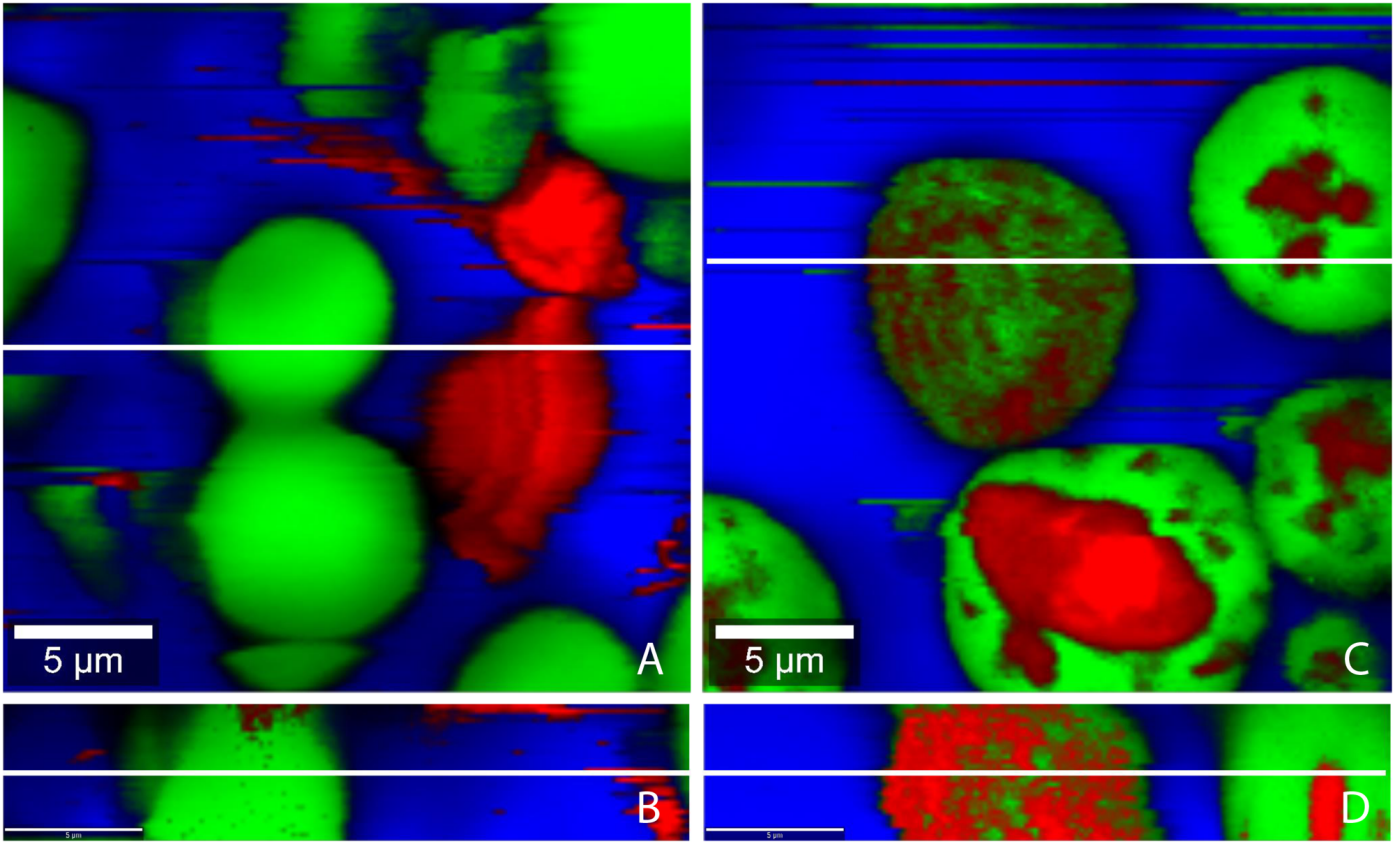
Confocal Raman microscopic colour-coded images of sunflower oil-in-water emulsion prepared by the different methods. (A, B) method 1, (C, D) method 2; (A, C) horizontal scans: 25x25 μm; 150x150 pixel; integration time: 0.13372 s; (B, D) depth scans: 25x5 μm; 150x30 pixel; integration time: 0.13372 s; white lines show position of the depth respectively horizontal scan.

**Fig 4:**
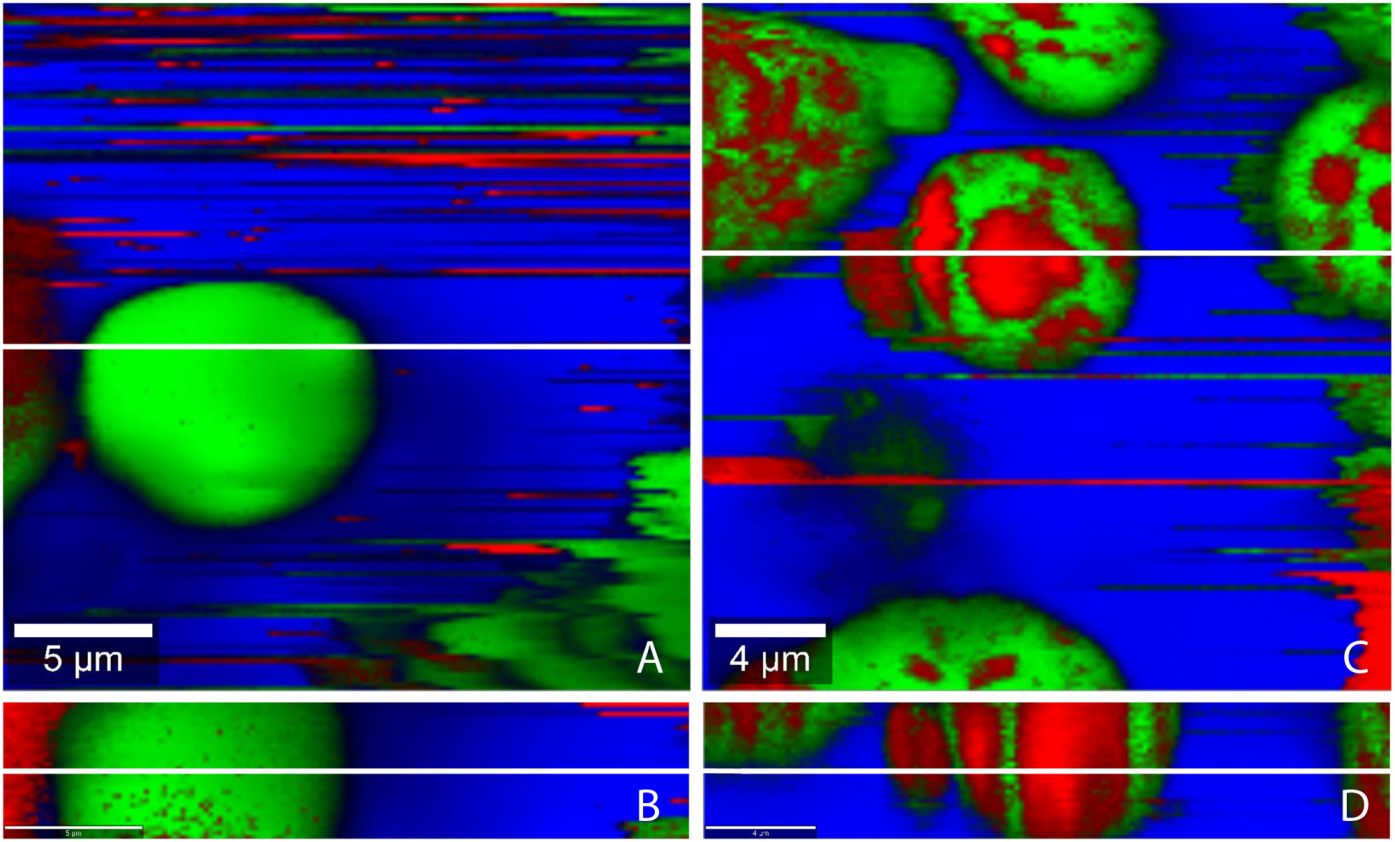
Confocal Raman microscopic colour-coded images of MCT-in-water emulsion prepared by the different methods. (A, B) method 1, (C, D) method 2; (A, C) horizontal scans: 25x25 μm; 150x150 pixel; integration time: 0.13372 s; (B, D) depth scans: 25x5 μm; 150x30 pixel; integration time: 0.13372 s; white lines show position of the depth respectively horizontal scan.

**Fig 5:**
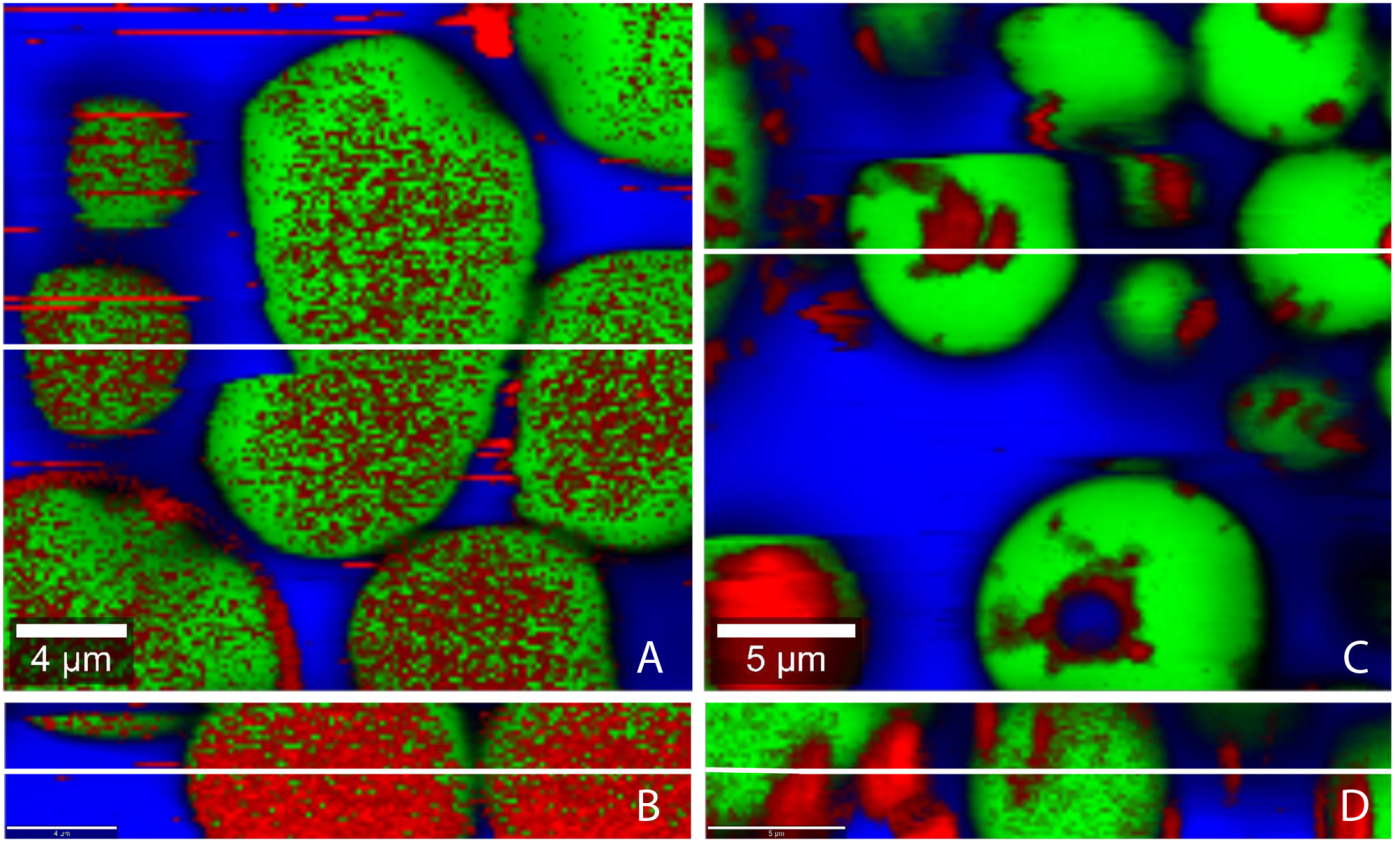
Confocal Raman microscopic colour-coded images of paraffin-in-water emulsion prepared by the different methods. (A, B) method 1, (C, D) method 2; (A, C) with; horizontal scans (A; C): 25x25 μm; 150x150 pixel; integration time: 0.13372 s; depth scans (B; D): 25x5 μm; 150x30 pixel; integration time: 0.13372 s; white lines show position of the depth respectively horizontal scan.

TE-particles are predominantly located in the water phase when emulsions with sunflower oil were prepared using method 1 (Fig 3A and 3B). Contrary, TE-particles are predominantly found in the oil phase when SFO/w emulsions were prepared by method 2 (Fig 3C and 3D). This means that the TE-particles remain in the phase, in which they had been dispersed prior to emulsification. Fig 4A, 4B, 4C and 4D show the spatial distribution of TE-particles in MCT/w emulsions prepared by either method. Again the TE-particles are mainly found in the phase in which they originally had been dispersed. Fig 5A, 5B, 5C and 5D show confocal Raman images of LLP-in-water emulsions. Surprisingly, here TE-particles can be found in oil and water phase, independent from the preparation method.

The different behavior of the medium polar SFO and MCT in comparison to the non-polar LLP can be explained by the different behavior of HPMC which is added to act as a polymeric o/w emulsifier. Depending on its affinity to the oil phase, HPMC forms a more or less densely packed interfacial film [26]. HPMC adsorbs strongly at the SFO/water and MCT/water interface and can thus effectively prevent that TE-particles move freely from oil to water or vice versa. In contrast, HPMC does not adsorb to the same extent at the LLP/water interface. Consequently, the resulting interfacial film is leaky and does not hinder the movement of the TE-particles. Therefore TE-particles partition according to their affinity between the two phases.

### Release of betulin from TE-oleogels

Release of TE was tested using Franz diffusion cells. Release experiments were conducted with o/w emulsions and oleogels as a reference. As betulin is the main constituent of the TE and the release properties of the other triterpenes are expected to be not substantially different, only the released amount of betulin was assayed.

**Fig 6:**
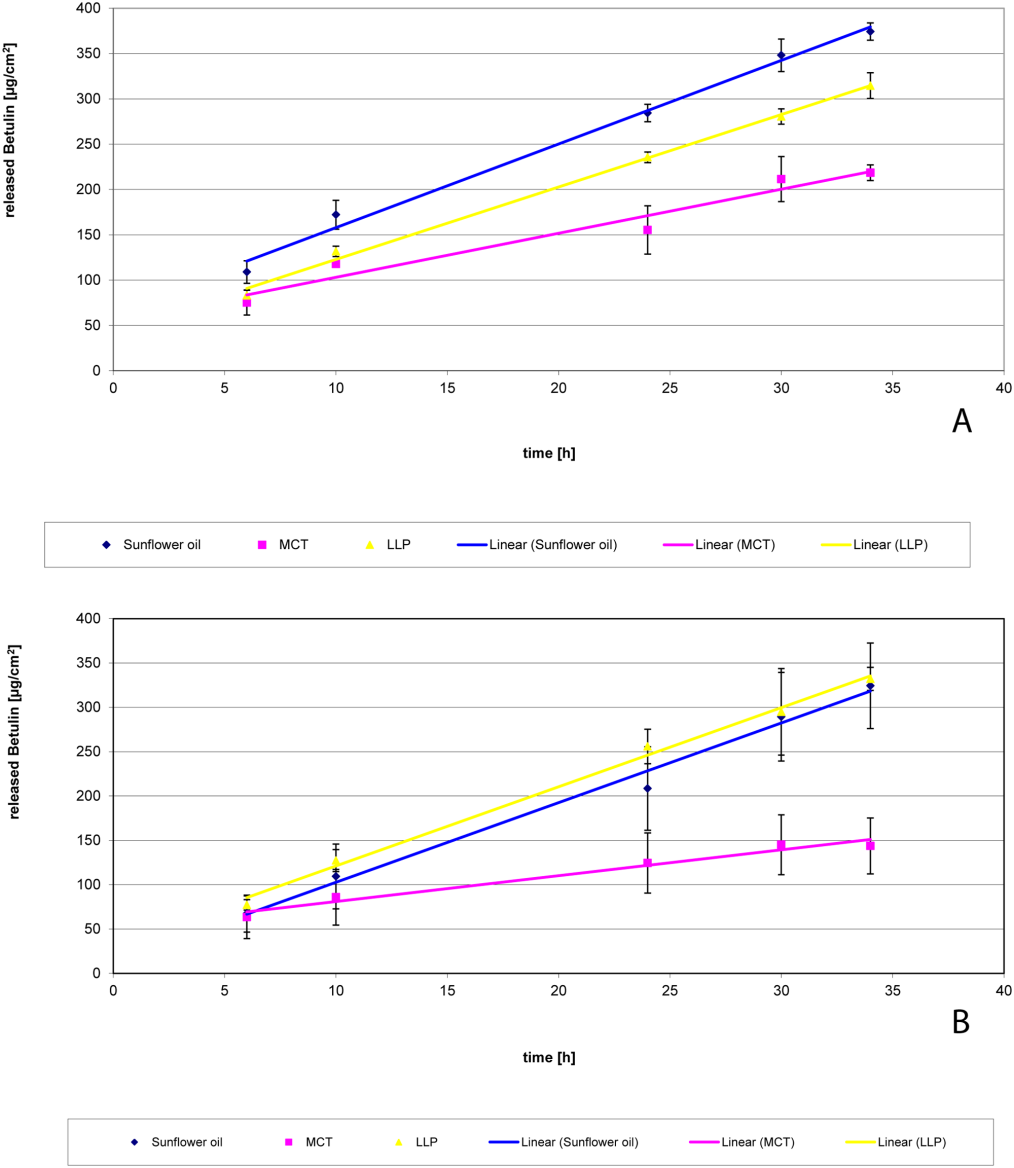
Release of betulin from oleogels with different concentrations of TE. (A) 2.0%, (B) 9.1% TE; n = 3; mean +/- standard deviation.

Fig 6 shows the release of betulin from oleogels. TE concentrations in the oleogels were 2.0% and 9.1% TE, respectively. These concentrations were chosen as (1) all emulsions contained 2.0% TE and (2) 9.1% oleogels (2 parts of TE dispersed in 20 parts of oil) were used for the preparation of emulsion by method 2. Release rate of betulin was linear in all cases independent from the oil type and the TE concentration. Release from SFO-oleogels and LLP-oleogels was clearly faster than from MCT-oleogels. The release rate of betulin from 2.0% oleogels was highest from SFO. Whereas no substantial difference between SFO and LLP was observed at the 9.1% level. Interestingly, the release from 2.0% oleogels is faster than from 9.1% oleogels. In order to allow to understand these findings, Tables 3 and 4 give some additional information about the amount of betulin in donor and receptor medium as well as the suspended fraction of TE in the oleogel and the oil/receptor partition coefficient of betulin. The dissolved quantities of betulin in the donor phases were calculated from the respective saturation solubility of betulin in the oil phase [40] and the applied amount of formulation. Furthermore, partition coefficients were calculated from the saturation solubility of betulin in the oil phase and in the receptor fluid (1010 μg/mL).

**Table 3:** Released amounts of betulin from oleogel 2.0% TE.

Oil phase	Initially dissolved amount of betulin in the oil phase [μg]	Amount of suspended particles in the oil phase [%]	Released amount of betulin in the acceptor medium after 34 h [μg]	Partition coefficient	Release rate [μg/cm^2^*h]	Release coefficient K_r_ [mL/cm^2^*h]
**Sunflower oil**	2836	82.3	756	2.8	9.228	3.25*10^−3^
**MCT**	5148	67.8	432	5.1	4.859	9.44*10^−4^
**Paraffin**	190	98,8	635	0.2	7.9911	4.20*10^−2^

Calculated amounts of triterpenes in donor and acceptor phase of the Franz diffusion cells and calculated distribution coefficient

**Table 4:** Released amounts of betulin from oleogel 9.1% TE.

Oil phase	Initially dissolved amount of betulin in the oil phase [μg]	Amount of suspended particles in the oil phase [%]	Released amount of betulin in the acceptor medium after 34 h [μg]	Partition coefficient	Release rate [μg/cm^2^*h]	Release coefficient K_r_ [mL/cm^2^*h]
**Sunflower oil**	2836	96.1	595	2.8	8.336	2.94*10^−3^
**MCT**	5148	92.2	295	5.1	2.9728	5.77*10^−4^
**Paraffin**	190	99.7	677	0.2	8.9893	4.73*10^−2^

Calculated amounts of triterpenes in donor and acceptor phase of the Franz diffusion cells and calculated distribution coefficient

In conclusion, these data show that release of betulin is lower from MCT than from SFO and LLP. This may explain why there is no strong positive effect of TE in MCT compared to MCT alone. For LLP, release would be comparable to SFO, however, LLP itself has a negative effect on wound healing which cannot be compensated by the addition of TE.

In principle, release might be dependent on several effects: TE concentration in the formulation [41], partition coefficient, viscosity, and the dissolution rate of the suspended fraction.

**Fig 7:**
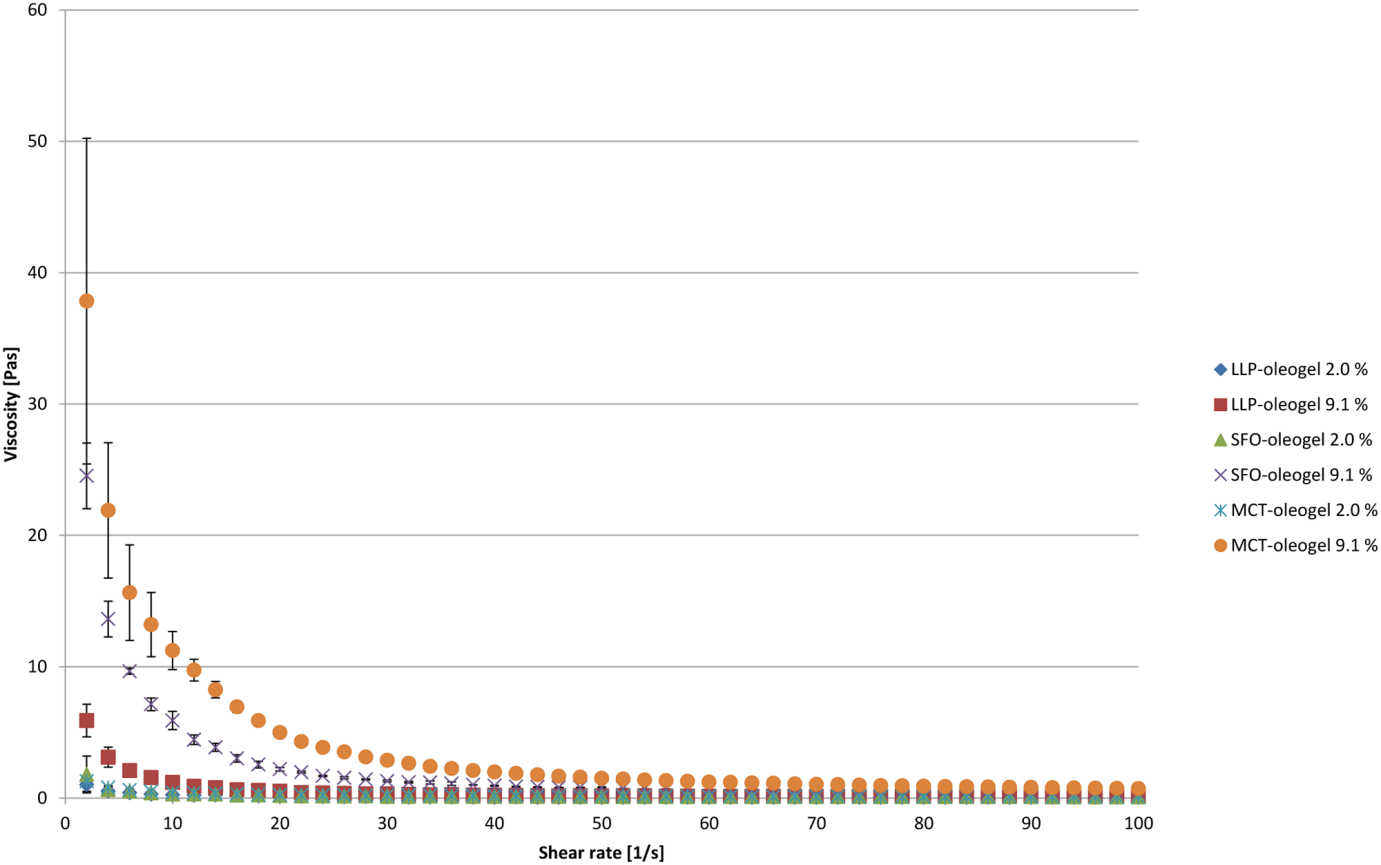
Flow curves (dynamic viscositiy vs. shear rate) of TE oleogels.

Betulin has an only limited solubility in all three oils tested and therefore at all TE levels investigated in this study (2%, 9.1%, and 10%) the majority (67.8–99.7%) of the incorporated powder (TE) is present as suspended particles having a specific surface area of 42 m^2^/g [42]. As the oil is saturated with betulin, its activity in all oleogels is 1 and the concentration and partition coefficient do not affect the release rate. The released amount of betulin after 34 h is lower than the initially dissolved amount of betulin in the oil when SFO and MCT are used. It exceeds the dissolved fraction when LLP is used as carrier. As the release rates from SFO and LLP are almost identical it can be concluded that the dissolution of the TE which is necessary for the release from LLP can also not be rate limiting. An explanation for the significantly lower release rate from MCT oleogels comes from the differences in viscosity, which is highest for these formulations (Fig 7). This is also reflected from the corresponding K_r_ values (Tables 3 and 4). Furthermore the viscosity is dependent on the TE content and thus explains the faster release from the 2.0% oleogel compared to the 9.1% oleogel. Consequently, the K_r_ of the 9.1% oleogel is 5.77x10^-4^ mL/cm^-2^h^-1^ whereas K_r_ of the 2.0% oleogel is 9.44x10^-4^ mL/cm^-2^h^-1^.

### Release of betulin from o/w emulsions

**Fig 8:**
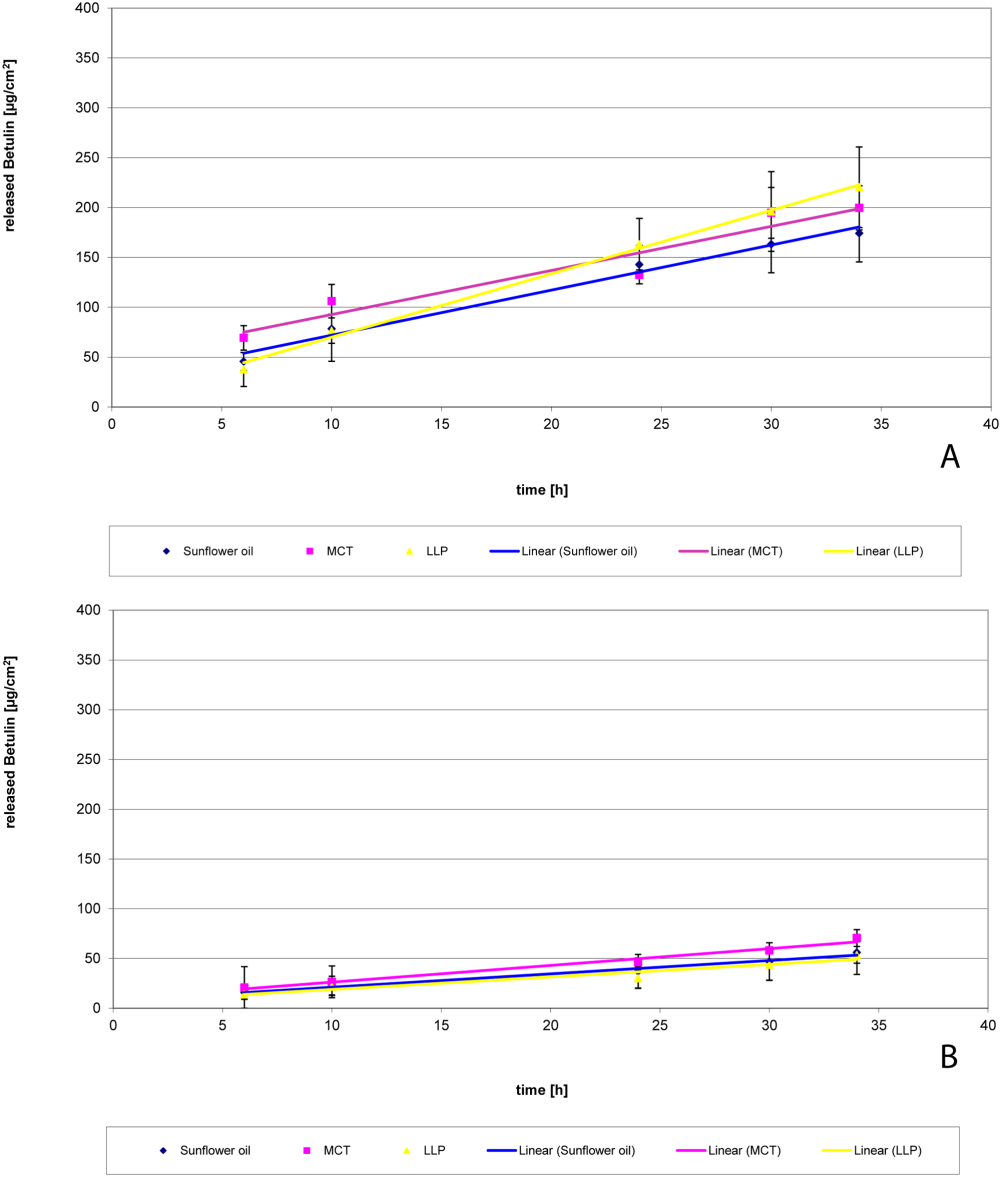
Release of betulin from o/w emulsions with 2% TE prepared with different methods. (A) method 1, (B) method 2; n = 3; mean +/- standard deviation.

Release of betulin from emulsions is shown in Fig 8. Release rate of betulin was linear in all cases independent from the preparation method indicating that release from infinite dose was diffusion controlled. Release from emulsions prepared by method 2 is significantly slower than release from emulsions yielding from method 1. But there is practically no difference between preparations with SFO, MCT and LLP.

Tables 5 and 6 give some additional information about the amount of betulin in donor and receptor medium as well as the suspended and dissolved fraction of TE in the emulsions. The later were calculated from the saturation solubility in water and the respective oil phases.

**Table 5:** Released amounts of betulin from emulsions prepared by method 1.

Oil phase	Initially dissolved amount of betulin in the oil phase [μg]	Dissolved amount of betulin in the water phase [μg]	Released amount of betulin in the acceptor medium [μg]	Amount of suspended particles in the formulation [%]	Release rate [μg/cm^2^*h]
**Sunflower oil**	567	0.06	410	96.1	4.5232
**MCT**	1030	0.06	409	92.9	4.4273
**Paraffin**	32	0.06	445	99.8	6.3686

Calculated amounts of triterpenes in donor (water and oil phase) and acceptor phase of the Franz diffusion cells.

**Table 6:** Released amounts of betulin from emulsions prepared by method 2.

Oil phase	Initially dissolved amount of betulin in the oil phase [μg]	Dissolved amount of betulin in the water phase [μg]	Released amount of betulin in the acceptor medium [μg]	Amount of suspended particles in the formulation [%]	Release rate [μg/cm^2^*h]
**Sunflower oil**	567	0.06	113	96.1	1.3434
**MCT**	1030	0.06	143	92.9	1.6851
**Paraffin**	32	0.06	108	99.8	1.2641

Calculated amounts of triterpenes in donor (water and oil phase) and acceptor phase of the Franz diffusion cells.

The released amount of betulin exceeds in all cases the dissolved amount in the outer aqueous phase of the emulsions by far. This makes it necessary that additional betulin is provided either from the suspended fraction in the aqueous phase or from dissolved fraction from the dispersed phase. When LLP is used as oil, neither the dissolved fraction in water nor the dissolved fraction in oil is high enough to deliver the released amount of betulin and dissolution of suspended particles is necessary.

As in all emulsions prepared by method 1 the TE-particles were suspended in the aqueous phase, the release rate is here almost independent from the oil used. As solubility in water is lower than in oil, the dissolution and the successive release were slower as compared to the oleogels. This explains why there was a better effect of the oleogels in the wound healing assays than the o/w emulsions.

Release rates from emulsions prepared using method 2 were again almost identical but much lower than from emulsions from method 1. This is especially surprising for the LLP-in-water emulsions because TE-particles have also been found in the aqueous phase even when they were prepared using method 2. Obviously the amount of TE in aqueous phase was not sufficient to deliver the required amount of betulin or the dissolution from these particles is hindered because they have been immersed in LLP. The slower release from SFO and MCT emulsions from method 2 can be explained by the effective surface area of the oil droplets which is lower than from the TE powder.

## Conclusion

Wound healing is a complex process and the effect of wound dressings is multifactorial. In case of TE-olegels, the effect of the plain oil phase as well as the release of the triterpenes (expressed as betulin release) both determine, whether wound healing is enhanced or impaired. Thus we have a complex triangular interaction between wound, vehicle and betulin.

From the above results it can be concluded that it is necessary to choose oil which does not substantially impair wound healing itself and to have a sufficiently high release of betulin to evolve its positive effect on wound healing. In this case the triterpenes can even compensate a slight negative effect like it was observed for plain SFO. Oleogels which release betulin not sufficiently, e.g. MCT, are not able to develop the desired activity even when the plain oil has a positive effect. Consequently, o/w emulsions which release betulin unsatisfactorily independent from the oil phase chosen are not recommended as a formulation option for wound healing. Even though we used TE and wound healing as model systems, our data can be extrapolated also to other experiments where oils are used as a vehicle, clearly demonstrating that their basic influence on the experiment has to be taken into account.
